# Synthesis of mangiferin derivatives, complexes, and carriers as potential therapeutic candidates for cancer treatment: an update

**DOI:** 10.3389/fphar.2025.1598719

**Published:** 2025-06-03

**Authors:** Elianny Melo-Betances, Crismery C. Rodríguez-Bautista, Alberto J. Núñez-Sellés

**Affiliations:** ^1^ Faculty of Sciences and Technology, Universidad Nacional “Pedro Henríquez Ureña” (UNPHU), Santo Domingo, Dominican Republic; ^2^ Research Division, Universidad Nacional “Pedro Henríquez Ureña” (UNPHU), Santo Domingo, Dominican Republic

**Keywords:** mangiferin, mangiferin derivatives, mangiferin complexes, mangiferin carriers, antitumor effect, bioavailability

## Abstract

Mangiferin (MF), a xanthonoid polyphenol, its derivatives, coordination and inclusion complexes, and carriers have demonstrated notable antitumor activity *in vitro* and *in vivo*. However, their clinical application remains limited due to MF’s poor water solubility and low systemic bioavailability. This review critically summarizes advances in the synthesis of MF derivatives and formulation strategies, such as metal complexes, cyclodextrin inclusion systems, and nanocarriers, developed over the past decade to enhance MF’s bioavailability and therapeutic efficacy. Promising results include glycosylated derivatives, MF-Se (IV) metal complexes, and β-cyclodextrin complexes, each contributing to improved solubility and cytotoxicity profiles. Continued research is essential to bridge the gap between experimental success and its clinical implementation in cancer therapy.

## 1 Introduction

Polyphenols, including xanthones, possess molecular characteristics that enable them to efficiently form chemical derivatives and inclusion complexes. These compounds are highly compatible with various carriers, which significantly enhances their water solubility and, consequently, their bioavailability (Ju et al., 2022). Incorporating these natural compounds has become an important area of research, particularly in exploring their potential as bioactive agents for several diseases (Ma et al., 2024). Mangiferin (MF), 1,3,6,7-tetrahydroxyxanthone-C2-β-D-glucoside, stands out as a powerful candidate for developing antitumor agents, targeting different types of cancers. This includes lung, brain, breast, cervix, and prostate cancers, as well as leukemia (Nuñez Selles et al., 2016; Iqbal et al., 2024). Based on the Biopharmaceutics Classification System, it is classified as a low solubility-low permeability compound (class IV) (FDACenter for Drug Evaluation and Research, 2017). The absorption of MF primarily occurs in the small intestine through passive diffusion. However, absorption rates can vary across gastrointestinal tract segments (Mei et al., 2021a). The GastroPlus software estimates its water solubility at 0.38 mg/mL (Khurana et al., 2017b). Furthermore, MF shows low intestinal permeability, likely due to its low lipophilicity (Kaliappan et al., 2015).

The synthesis of MF derivatives and complexes aimed at improving bioavailability has seen limited progress over the past decade. Liang et al. (2019) reported the complexation of MF with a polyamine-modified β-cyclodextrin (PA-CD), a polysaccharide composed of glucose units linked by α-1,4-glycosidic bonds. PA-CD features a unique structure that includes a hydrophobic inner cavity and a hydrophilic exterior, allowing it to form an inclusion complex that improves both bioavailability and cytotoxicity compared to free MF. Additionally, copper (II) and zinc (II) complexes of MF have shown increased cytotoxicity relative to free MF, likely due to their ability to intercalate with DNA and inhibit topoisomerase (Qin et al., 2016).

The synthesis of these metal complexes involves reacting MF with metal salts under specific conditions to produce stable, water-soluble complexes. Furthermore, Núñez-Sellés et al. (2022) found that MF-selenium (IV) complexes provided greater protection against protein degradation and demonstrated lower peroxidation potential than MF-Cu (II), and Zn (II) complexes, suggesting the potential advantages of using MF-Se (IV) complexes for cancer treatment. Recent efforts to enhance the bioavailability of new MF formulations have explored various types of carriers. These include organic carriers such as nanoparticles, lipid-based carriers, protein-based carriers, and polymer-based carriers. Inorganic carriers like mesoporous silica and gold nanoparticles have also been explored (Barakat et al., 2022; Vishwakarma et al., 2024). This review focuses on the advances made in the last decade in synthesizing MF derivatives, complexes, and carriers as potential antitumor agents.

## 2 Data search

Published reports on MF were downloaded and reviewed from specialized data sources, including PubMed/MedLine, ScienceDirect, Google Scholar, SciFinder, and the TRIP Database. The search methodology include terms as “mangiferin,” “mangiferin derivatives,” “mangiferin complexes,” “mangiferin carriers,” “mangiferin complex synthesis,” “mangiferin bioavailability,” “mangiferin permeability,” “mangiferin pharmacological effects” “mangiferin antitumor effects,” “Inclusion criteria focused on studies published between the years 2015–2024, emphasizing comprehensive data regarding bioavailability, mechanisms of action, and other research related to the chemical synthesis and antitumor effects of MF, its complexes, and carriers on cancer. The search included *in vitro*, *in vivo*, and clinical studies, while reports on skin treatment formulations and cosmetic applications were excluded and will be discussed in a separate context.

## 3 Antitumor effects of mangiferin

The potential effects of MF as an antitumor agent have been discussed elsewhere by several authors (Mei et al., 2021b; Sarfraz et al., 2023). One of the major mechanisms through which MF exhibits its anticancer and apoptosis-inducing effects is through the inhibition of the NF-ΚB pathway and its antioxidant effects at the cellular level. Nuclear translocation of NF-kB has induced the transcription of several genes involved in various types of cancer, including brain, breast, lung, and gastric cancer (Moneva-Sakelarieva et al., 2025). NF-kB activation and cell proliferation can activate the autocrine production of TNFα, leading to increased NF-kB activation and resistance to apoptosis. Inflammation plays a pivotal role in all stages of the development and progression of cancer; cancer cells release several cytokines and chemokines, which are related to immune-related tumor progression, with increased inflammation (Rahmani et al., 2023). On the other hand, OS may increase the inflammatory environment that promotes tumor growth and metastatic potential (Yu et al., 2022). Therefore, when exploring the potential role of MF and its derivatives, complexes, and carriers for enhancing cancer treatment, these two effects (anti-inflammatory and antioxidant) have a significant influence on its antitumor effects.

## 4 Enhancement of antitumor effects of mangiferin

### 4.1 Mangiferin derivatives

**FIGURE 1:**
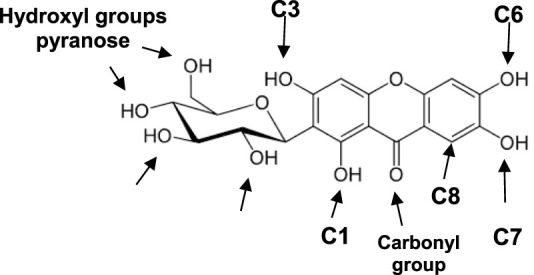
Chemical structure of mangiferin (MF). Arrows indicate possible reaction sites, depending on the synthetic pathway to produce MF derivatives and/or complexes.

MF contains two hydroxylated aromatic rings, with four hydroxyl groups located on carbons 1, 3, 6, and 7; a xanthone ring, which includes a carbonyl group, and a glucose moiety (pyranose group) attached to carbon 2 (Figure 1). Consequently, the hydroxyl groups on C3, C6, and C7 are the primary target sites for synthesizing MF derivatives. Some substitution reactions may involve the hydrogen atom on C8, as well as C-C enzymatic cleavage at C2. The hydroxyl group on carbon atom C1 is hindered by steric effects from the pyranosyl group and forms an intramolecular hydrogen bond with the adjacent carbonyl group (Gómez-Zaleta et al., 2006). However, in a strongly basic medium, it may be possible to bond the oxygen atom of the C1-hydroxyl group.

**FIGURE 2:**
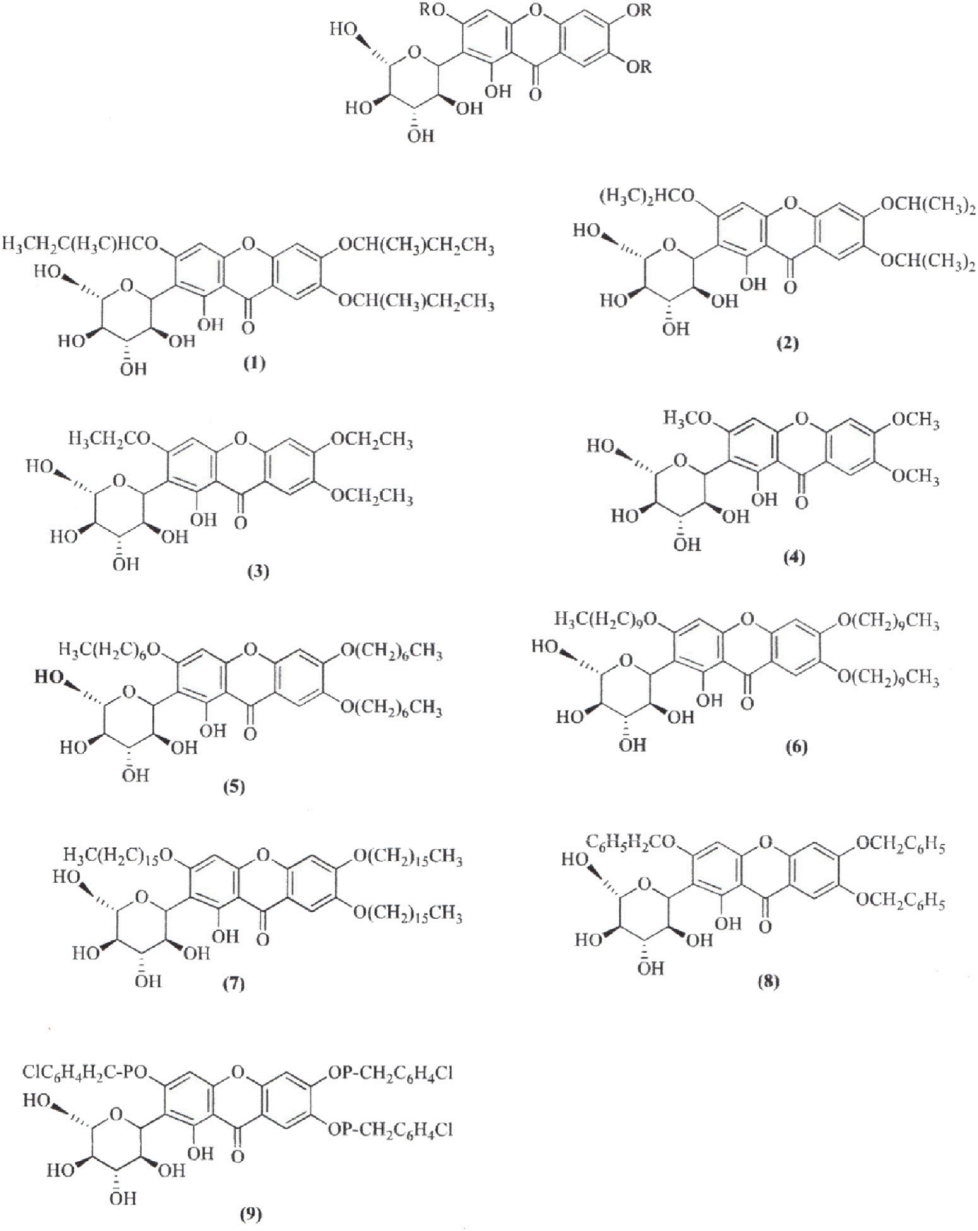
Chemical derivatives to enhance the mangiferin (MF) antidiabetic activity by inhibiting the Protein Tyrosine Phosphatase 1B (PTP1B). Reaction conditions: DMF as solvent; K_2_CO_3_, RX, stirring 10 h, 60°C. Derivatives 6 and 9 showed 100% and 62.5% higher inhibition than MF against PTP1B (Turkar et al., 2024).

**FIGURE 3:**
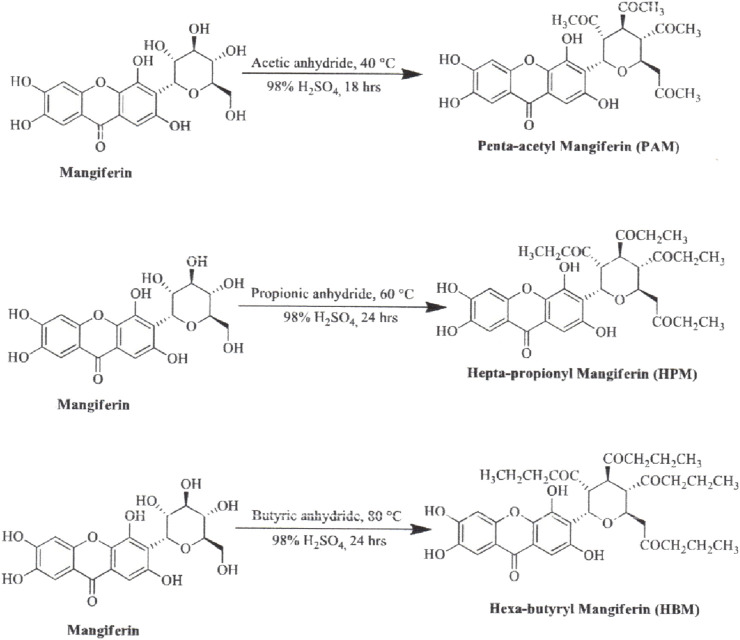
Chemical derivatives to enhance the mangiferin (MF) antidiabetic activity in a streptozotocin-induced hyperglycemia mouse model. Reaction conditions: alkyloxy anhydride, H_2_SO_4_, stirring 18 h, 40°C. All derivatives showed significantly higher antidiabetic activity than MF (Turkar et al., 2024).

**FIGURE 4:**
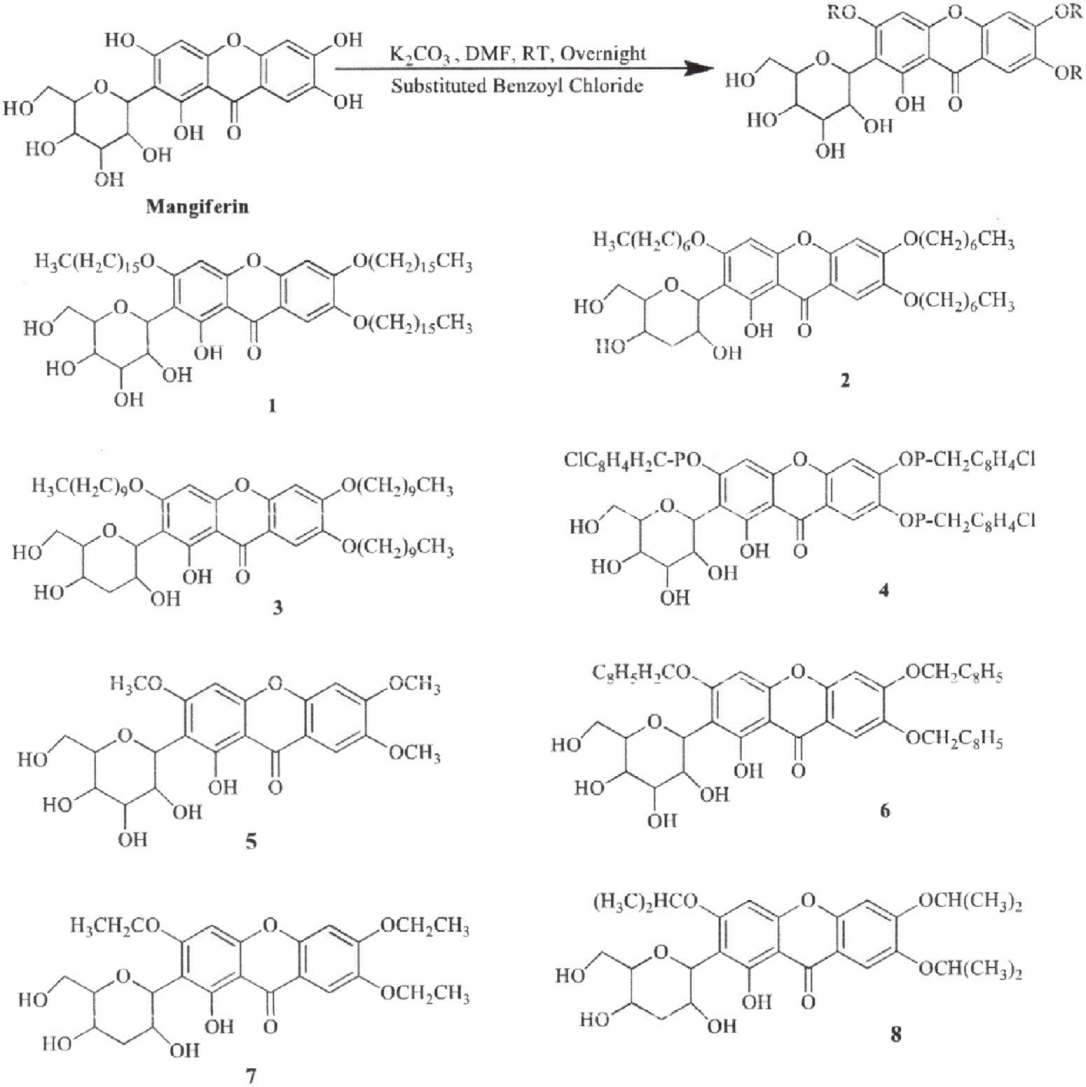
Chemical derivatives to enhance the mangiferin (MF) analgesic activity by inhibiting the cyclooxygenase enzyme. Reaction conditions: DMF as solvent, K_2_CO_3_, stirring overnight, room temperature. MF and all its derivatives had similar analgesic and anti-inflammatory effects (Turkar et al., 2024).

The synthesis of MF derivatives using alkyl halides at 60°C, with dimethylformamide (DMF) as the solvent at pH = 8, has led to the formation of nine derivatives, as illustrated in Figure 2 (Turkar et al., 2024). Notably, the substitution with a decyloxy group at positions C3, C6, and C7 (derivative 6 in Figure 2) exhibited the highest inhibition ratio (100%), compared to MF, indicating a significant enhancement in antidiabetic activity due to this novel derivative. Similarly, a positive outcome was observed with acetyl derivatives, which were created by esterifying the four hydroxyl groups in the pyranosyl group. This method produced acetic, propionic, and butyric derivatives that demonstrated greater antidiabetic activity than MF (Figure 3). Additionally, various modifications such as acetylation, benzylation, cinnamoylation, and methylation were conducted to develop different MF derivatives aimed at increasing antioxidant activity (Figure 4). The antioxidant capabilities of the synthesized derivatives were assessed through the DPPH test and by evaluating the inhibition of lipid peroxidation. The results indicated a significant improvement in the antioxidant activity of the acetylated MF derivative (compound 2, Figure 4).

**FIGURE 5:**
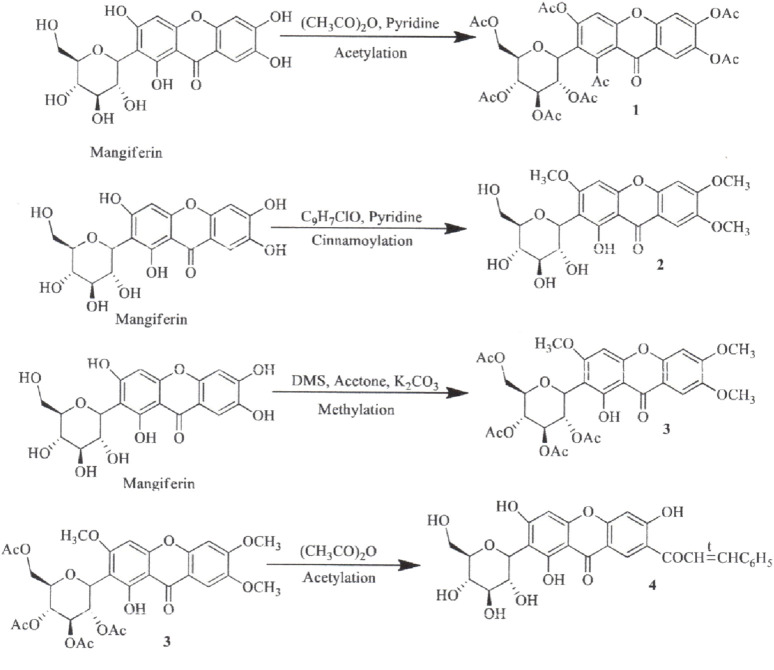
Chemical derivatives to enhance the antioxidant activity of mangiferin (MF) by DPPH test and inhibition of lipid peroxidation. Reaction conditions: Acetylation and cinnamoylation (alkyloxy and pyridine as solvent); Methylation (acetone, DMS, K_2_CO_3_, stirring between 7 and 36 h, room temperature. Derivatives 3 and 4 had higher antioxidant activity than MF by 77.2%, and 83.7%, respectively (Turkar et al., 2024).

**FIGURE 6:**
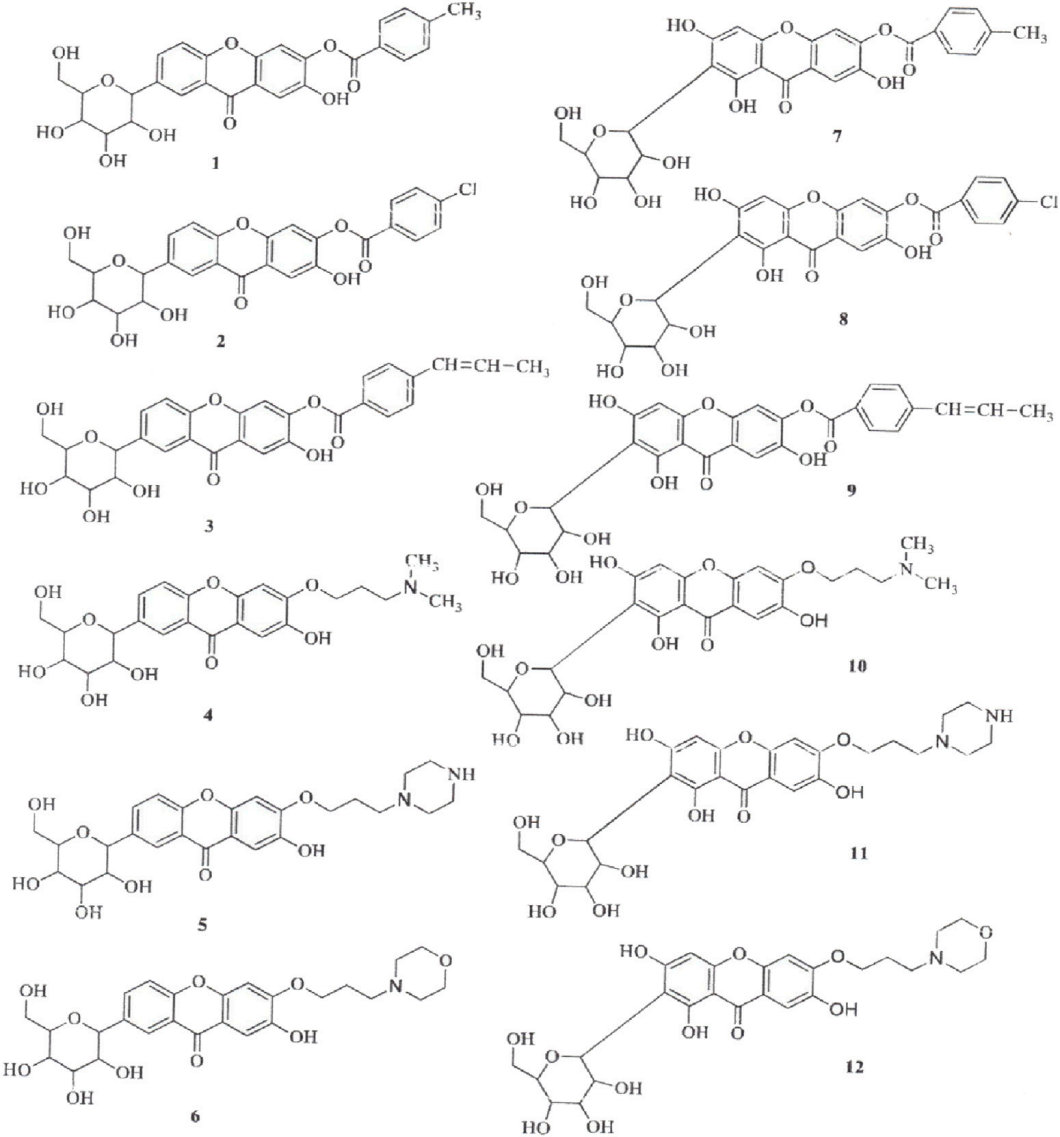
Chemical derivatives to enhance the antitumor activity of mangiferin (MF) in breast cancer cell line (MDA-MB-231). Derivatives 6, 11, and 12 showed significantly higher cytotoxic effects than MF in this cell line (Patil et al., 2022).

The synthesis of aryl and alkyl halide MF derivatives has been reported to enhance its analgesic properties (Khare and Shanker, 2016). However, no significant differences in analgesic effects were observed (Figure 5). Patil et al. (2022) reported the synthesis of novel esterified and alkylated aryl amine derivatives of MF aimed at improving its *in vitro* antioxidant and antitumor effects (Figure 6). Some of these derivatives had higher cytotoxic effects than MF against the breast cancer cell line MDA-MB-231. Liu et al. (2017) found comparable results using xanthones in the same cancer cell line.

The synthesis of glycosylated derivatives of MF is regarded as a promising strategy for enhancing its biological effects, although it has not been extensively documented in the literature. One approach involved creating MF-fructosyl derivatives through biotransformation using *Arthrobacter nicolianae* and dextrasucrase, specifically targeting the C6 position of the pyranosyl group (He et al., 2015; Septiana et al., 2020). This method was associated with claims of treating antitumor-related diseases. In a similar effort to increase the antioxidant activity of MF, researchers used a recombinant maltogenic amylase to produce MF-glucosyl-α-(1→6)-MF and maltosyl-α-(1→6), resulting in a 5500-fold increase in water solubility compared to MF. Nevertheless, the MF glucosides exhibited similar DPPH free radical scavenging activity (Wu et al., 2021). Additionally, Lee et al. (2022) reported the synthesis of an MF-glucosyl-α-(1→4) derivative through glucosyl transferase from *Thermoanaerobacter* sp. This derivative was formulated with β-cyclodextrin to create an inclusion complex, achieving a remarkable 5093-fold increase in water solubility and demonstrating significantly higher anti-inflammatory activity than MF.

**FIGURE 7:**
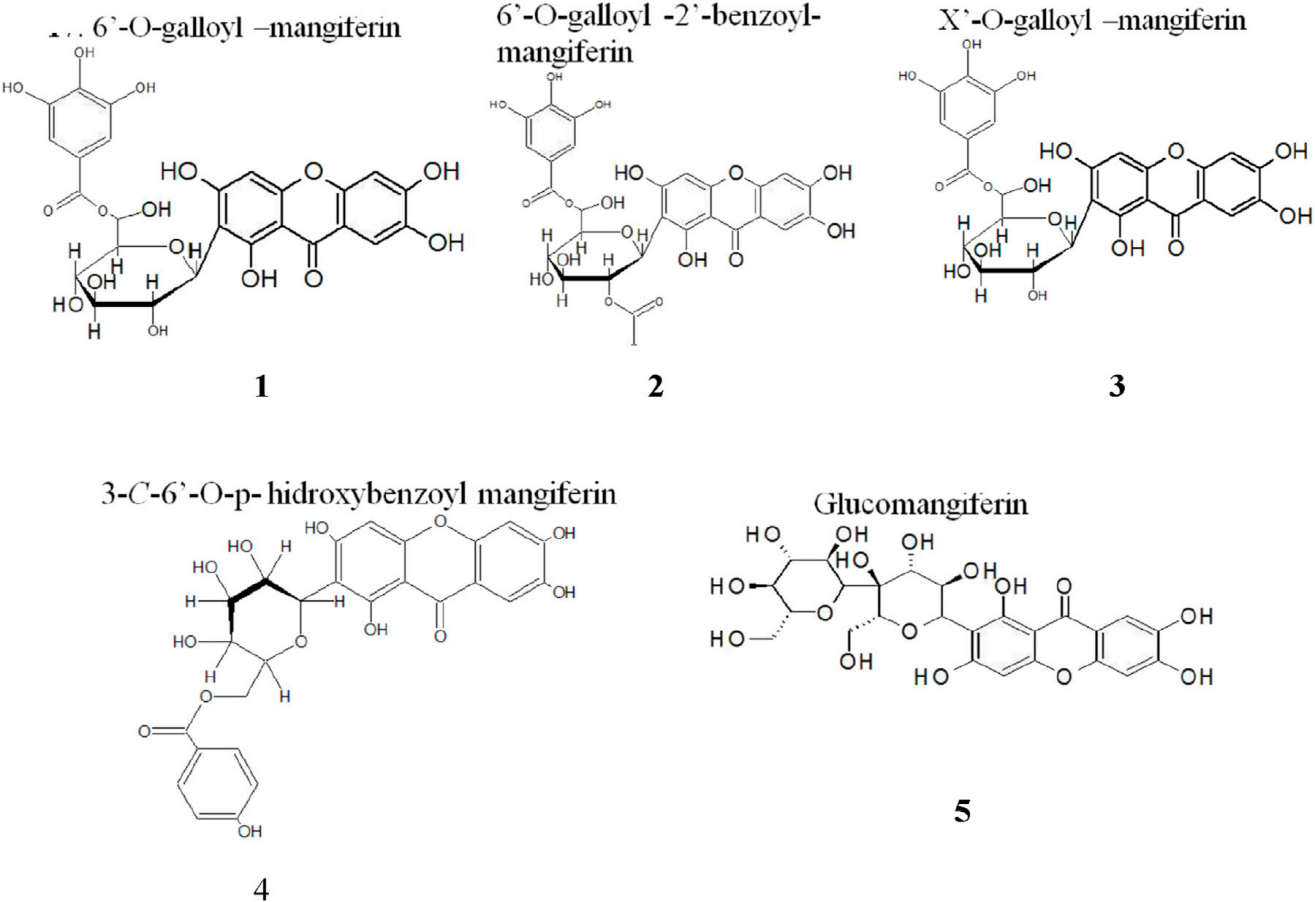
Mangiferin derivatives found in aqueous mango stem bark extract by HPLC/MS-MS (Núñez-Sellés et al., 2020).

Five new derivatives of MF were identified in the extract of mango stem bark, which have not been documented previously (Figure 7) (Núñez-Sellés et al., 2020). The underlying hypothesis suggests that the biological effects observed in plant extracts containing MF may result from a synergistic combination of MF, glycosylated MF derivatives, galloylated MF derivatives, and benzoylated MF derivatives. Moreover, computational techniques have been employed to profile the interaction of MF at the atomic level against nine selected molecular targets with clinical relevance in tumorigenesis. In an attempt to investigate the potential of MF as a viable starting point for synthetic exploration of MF-based analogs, extensive structural modifications have been explored, which need to be realized experimentally (Taiwo et al., 2018). Several studies have been conducted on QSAR studies for MF derivatives focused on antimicrobial (Nortje et al., 2025) or antidiabetic (da Silva-Lopes et al., 2024), but there is a lack of information about the antitumor effects of MF derivatives.

In summary, alkylation and acylation reactions at positions C3, C6, and C7 resulted in MF derivatives with improved antidiabetic and antioxidant activities, respectively. Additionally, esterification and aryl-alkylation reactions, particularly at position C6, produced MF derivatives that exhibited greater cytotoxicity against a breast cancer cell line compared to free MF. However, not all MF derivatives synthesized through these chemical reactions demonstrated improved biological activity over MF, which underscores the necessity for quantitative structure-activity relationship (QSAR) studies focused on the biological effects of MF derivatives (Benard and Chi, 2015). A promising strategy for developing antitumor agents from MF includes creating derivatives with oxygenated radicals, such as acetylated, benzoylated, glycosylated, and galloylated, attached to both the xanthone ring and the pyranosyl group.

### 4.2 Mangiferin complexes

#### 4.2.1 Inclusion complexes with cyclodextrins

**FIGURE 8:**
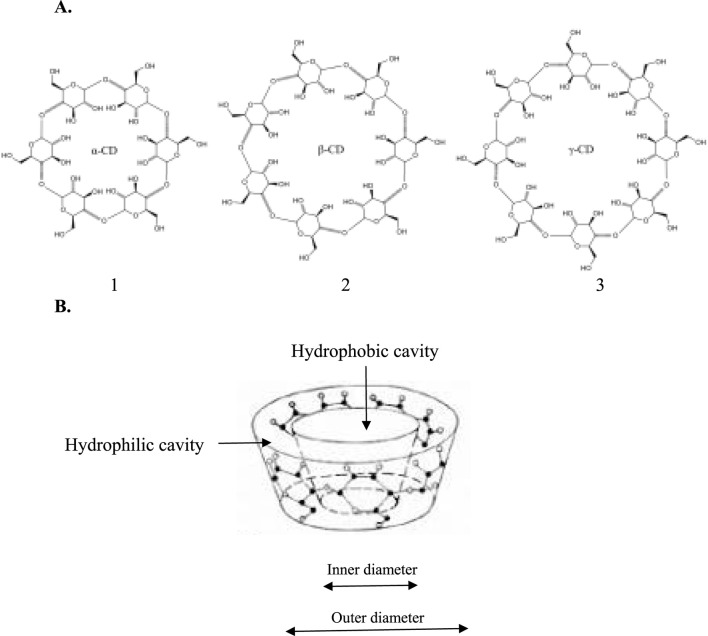
Structures of the most common cyclodextrins (CyDs) to enhance the bioavailability of poorly water-soluble bioactives. 1. α-cyclodextrin; 2. β-cyclodextrin; and 3. γ-cyclodextrin (Sharma and Baldi, 2016). **(A)** 2D structures; **(B)** 3D toroidal structure of CyDs.

Cyclodextrin (CyD) inclusion complexes are one of the strategies for increasing the solubility of poorly soluble drugs. CyDs belong to the family of cyclic oligosaccharides, with the primary being α-, β-, and γ-CyD, which consist of 6, 7, and 8 units of glucopyranose, respectively (Figure 8A) (Crini et al., 2018). Due to their toroidal or truncated cage-like supramolecular configurations (Figure 8B), they can encapsulate hydrophobic compounds by forming inclusion complexes (Alshati et al., 2023). Various chemical modifications, such as methylated β-CyD (Santos et al., 2017), hydroxypropyl β-CyD (D’Aria et al., 2022), and hydroxypropyl methyl β-CyD (Zucca et al., 2025) have envisaged the use of CyDs in a range of pharmaceutical and medical applications. Drug inclusion complexes, particularly those involving β-CyD, have demonstrated improved solubility, enhanced bioavailability, reduced drug resistance, target delivery, and better tissue or organ penetration (Carneiro et al., 2019).

Several studies have reviewed the use of cyclodextrins (CyDs) to enhance the bioavailability and membrane permeation of antitumor drugs (Gidwani and Vyas, 2015; Gandhi et al., 2020). Notable examples of drugs that form inclusion complexes with CyDs include oxaliplatin (Zhang et al., 2016), 5-fluorouracil (Di Donato et al., 2016), bufalin (Zou et al., 2017), gemcitabine (Rescifina et al., 2019), and bicalutamide (De Gaetano et al., 2022), among others. Additionally, the use of CyDs to deliver natural compounds with antitumor activity has also been reviewed (Christaki et al., 2023).

Notably, only a few reports over the last decade have focused on the inclusion complexes of MF for pharmaceutical applications. It highlights the need to further explore this research approach to enhance MF bioavailability. Hernández-García et al. (2022) documented the physicochemical characteristics of the MF-β-CyD inclusion complex, showing that MF was incorporated into the inner cavity of the β-CyD toroid through the xanthone ring, forming a stable 1:1 stoichiometric inclusion complex (MF-β-CyD). Li et al. (2021) reported the same sterical behavior for MF-γ-CyD, regarding the inclusion of the xanthone ring into the inner cavity, which enhanced the antioxidant activity of MF. Additionally, the use of polyamine-β-CyD improved the formation of another inclusion complex (MF-polyamine-β-CyD) with the same 1:1 stoichiometric ratio, demonstrating significantly lower toxicity to human normal cells compared to free MF (Liang et al., 2019).

#### 4.2.2 Other inclusion complexes

Inclusion complexes of MF with a mixture of humic acids can significantly increase its water solubility fivefold, from 0.02 mg/mL up to 0.1 mg/mL (Morozova et al., 2022). These nanoparticles (HA-MF) may function as lipophilic and pH-responsive drug carriers that can target cancer cells by inducing apoptosis and causing cell cycle arrest, in addition to their proven antiviral effect. Targeted polymeric self-assembled nanoparticles with hyaluronic acid, a naturally occurring glycosaminoglycan found throughout the body’s connective tissue, have been developed to deliver MF with a high loading content of 6.86% ± 0.60%. These nanoparticles demonstrate excellent blood circulation and exhibit missile-like delivery to the pancreas (Wang M. et al., 2022).

#### 4.2.3 Metal coordination complexes

Metal coordination of phenolic compounds has been widely described in the literature (Kalinowska et al., 2020). Metals can alter biological activity, including the biological properties of ligands, by affecting the molecular structure and charge density of phenolic compounds. Research has shown that metal chelates can have higher pharmacological effects than the phenolic compounds alone (Kowalczyk et al., 2021). Research on the therapeutic applications of coordination complexes with bioactive organic ligands has shown significant progress during the first 2 decades of the 21st century (Khater et al., 2019). The redox activities of these complexes and their impact on homeostasis at the cellular level have been extensively studied (Kasprzak et al., 2015). The use of flavonoids as bioactive ligands for synthesizing metal complexes focused on 4′,7,8-trihydroxy-isoflavone combined with zinc (II), copper (II), manganese (II), nickel (II), cobalt (II), and selenium (II) has been studied by Tang et al. (2011). The results showed that all the metal complexes exhibited a higher cytotoxic effect than the free isoflavone. The metallic nucleus was coordinated with the two adjacent hydroxyl groups in the catechol moiety of two isoflavone derivative ligands, which had a strong interaction with calf-thymus DNA.

Fan et al. (2017) reported on the use of epigallocatechin gallate-iron (III) complexes as a drug delivery system aimed at enhancing conventional cancer treatments. These complexes were found to reduce cancer metastasis by eliminating epithelial-mesenchymal transition cells. Additionally, Xie et al. (2021) reviewed other applications of metal-polyphenol complexes (MPN) in cancer therapy and diagnosis with several polyphenolic ligands (myricetin, quercetin, luteolin, fisetin, and epigallocatechin) and metals such as Fe (II and III), Mg (II), Mn (II), Al (III), Ti (IV), Co (II), V (III), Cu (II), Zn (II), Ni (II), Cr (IV), Zr (IV), and Mo (IV), including also several transition metals, which have been explored as chemotherapeutics or diagnostic tools in cancer research. These MPN complexes are being intensively studied because of their biomedical applications (Rosenblum et al., 2018). However, synthetic routes and biomedical applications of MF metal complexes have not been extensively studied. Exploring the potential of forming metal complexes to enhance the antitumor mechanism of MF could be a promising approach to increase its bioavailability and bioactivity (Rodríguez-Arce and Saldías, 2021).

**FIGURE 9:**
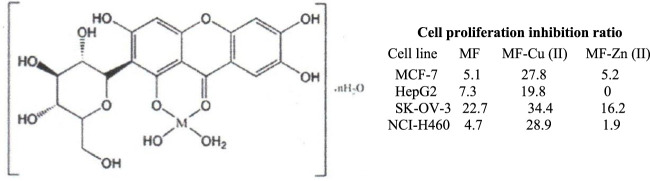
Proposed structure of the mangiferin (MF)-metal complex -Cu (II) and Zn (II)- with improved inhibition of cell proliferation in cancer cell lines as compared to MF (MTT assay) (Qin et al., 2016).

Qin et al. (2016) have reported the synthesis of MF-Cu (II) and MF-Zn (II) complexes in a 1:1 stoichiometric ratio, at pH 7.5, and proposed the structure shown in Figure 9 according to the spectral data. MF was attached to the metal nucleus through the electron pair of the oxygen atom in the carbonyl group and the hydroxyl group in C1. MF-metal complexes were tested through the MTT test on breast (MCF-7), liver (HepG2), ovarian (SKOV 3), and lung (NCI-H460) cancer cell lines. Inhibition of cell proliferation was enhanced between 1.6-fold and 6.1-fold, as compared to MF. MF-Cu (II) complex showed the best inhibition number for MCF-7 and NCI-H460 cancer cell lines, and the poorest for SKOV3 cell line. The MF-Zn (II) complex did not show a significant improvement in cell proliferation as compared to MF.

The formation of metal complexes with MF in plant aqueous extracts may help explain why the biological activity of these extracts can sometimes exceed that of the isolated MF (Nuñez Selles et al., 2016). It has been suggested that the combination of MF with trace elements such as Cu (II), Zn (II), and Se (IV)—likely through the formation of MF metal complexes—may contribute to the synergistic effects of both MF and the inorganic elements. Enhancing the antitumor effect of MF by forming metal complexes could potentially increase both its bioavailability and bioactivity. *In vitro* studies have shown that MF-Se (IV) complexes in stoichiometric ratios of 1:1, 2:1, and 3:1 exhibited greater inhibition of lipid peroxidation and stronger protection against protein oxidation compared to similar complexes formed with Cu (II) and Zn (II) (Núñez-Selles et al., 2022). Selenium, as the central nucleus of MF or polyphenol metal complexes, has not been extensively studied, despite its attractive biological properties (Kiełczykowska et al., 2018; Zoidis et al., 2018). Table 1 summarizes several types of MF inclusion and metal complexes that have enhanced the antitumor effects of MF through their increase in water solubility and cell permeability, which have been reported in the last decade (2015–2024).

**TABLE 1:** Chemical derivatives and complexes of mangiferin (MF) and other antitumor drugs to enhance its water solubility and cell permeability.

Type	Antitumor agent	Ligand	Dose	Enhanced effect	References
Chemical derivatives	MF-decyl	Alkyl	5.4 μM	↑PTP1 B inhibition (100%)	Turkar et al. (2024)
	MF-alkyl/aryl	Arylalkyl	0.35 M	≈Analgesic activity	Khare and Shanker (2016)
	MF-acetyl	Alkyloxy	0.5 mM	↑Hypoglycemic activity	Turkar et al. (2024)
	MF--derivatives	Galloyl- & Hydroxybenzoyl	0.1 mM	Synergic effect	Núñez-Sellés et al. (2020)
	MF-Glycosyl	Glycosyl	8–10.5 μM	↑Antioxidant activity	He et al., 2015; Septiana et al., 2020; Lee et al., 2022
Inclusion complexes	Oxaliplatin	β-CyD	31–45 μM	↑Cytotoxicity on HCT 116 and MCF-7 cells	Zhang et (2016)
	5-fluorouracil	β-CyD	18–55 μM	↑Cytotoxicity on MCF-7, Hep G2, Caco-2, A-549 cells	Di Donato et al. (2016)
	bufalin	β-CyD	500 nM	↑Cytotoxicity on HCT116 cells	Zou et al. (2017)
	gemcitabine	β-CyD	0.5 μM	↑Cytotoxicity on $-549 cells	Rescifina et al. (2019)
	bicalutamide	β-CyD	0.2 mM	↑Cytotoxicity on PC-3 and DU-145 cells	De Gaetano et al. (2022)
	NA	Non-modified and modified β-CyDs	NA	Several biological effects	Santos et al. (2017) Hernández-García et al. (2022) D’Aria et al. (2022) Zucca et al. (2025)
	MF	Hyaluronic acid	NA	↑Cytotoxicity MTX on MCF-7 cells	Wang et al. (2023)
	MF	Polyamine modified β-cyclodextrin	NA	↓Toxicity in human normal cells (HEK 293)	Liang et al. (2019)
		Humic acids	NA	↑Apoptosis induction	Morozova et al. (2022)
Metal complexes	Metal complex	Epigallocatechin gallate	35–70 μM	↓Cancer metastasis	Fan et al. (2017)
		MF	2.3 μM	↑Cytotoxicity on MCF-7, Hep G2, SKOV3, and NCI-H460 cells	Qin et al. (2016)
		MF	8–16 μM	↑Protein protection and ↓lipid peroxidation	Núñez-Sellés et al. (2022)
		Myricetin, quercetin, fisetin, luteoin and epigallocatechin	NA	↑Cytotoxicity on several cancer cell lines	Xie et al. (2021)

Legend: PTP1B: Protein tyrosine phosphatase 1B; β-CyD: β-Cyclodexdtrin; MF: mangiferin; MTX: methotrexate; NA: not applicable.

## 5 Mangiferin carriers

### 5.1 Organic carriers

Nanoparticle carriers are considered one of the most promising methods for enhancing the bioavailability of MF in cancer treatment, as extensively reviewed by Barakat et al. (2022). Among these carriers, Nanostructured Lipid Carriers (NLCs) represent a second generation of lipid-based carriers developed to address the limitations of earlier solid lipid carriers (SLCs) (Viegas et al., 2023). NLCs consist of a mixture of biocompatible solid and liquid lipids (in a 7:3 ratio), along with a surfactant, offering higher drug loading capacity and stability compared to SLCs. The MF-NLC formulation has been utilized for ocular delivery, as reported by Santonocito et al. (2022), using 888ATO as the solid lipid and miglyol as the liquid lipid. This formulation was found to be non-irritating to the eyes and demonstrated a significant increase in the antioxidant activity of MF. Khurana et al. (2017a) prepared MF-NLC by refluxing various molar ratios of MF and Phospholipon 90G (1:1, 1:2, 1:3) before incorporating them into the NLC formulation, which consisted of Compritol and Labrafil M2125. They achieved a controlled release formulation that lasted up to 10 h, resulting in a fivefold increase in MF plasma concentration. Additionally, MF-SLC was successfully developed using Labrafil M 2130 CS as the lipid carrier and Tween 80 as the surfactant, which enhanced antidiabetic activity in Wistar rats with streptozocin-induced diabetes (Foudah et al., 2024).

A noteworthy approach in the treatment of lung cancer has involved the use of a system based on transferrin (Tf) and MF, capitalizing on the overexpression of Tf in lung cancer cell receptors (Zhou et al., 2023). The Tf-MF-SLN was created using the emulsification-solvent evaporation method with DSPE-PEG2000-Tf (where DSPE stands for 1.2-Distearoyl-sn-glycero-3-phosphoethanolamine). The release of MF, MF-SLN, and MF-SLN-Tf was investigated in a specific lung cancer cell line using confocal microscopy. It was observed that MF-SLN-Tf was internalized by the cells within 2 h, whereas free MF and MF-SLN were not detected. Additionally, the benefits of MF-SLN-Tf were evident in terms of cell cytotoxicity and the inhibition of cell proliferation.

Nanoemulsions serve as versatile carriers for the delivery of lipophilic, hydrophilic, and amphiphilic bioactives, enhancing the bioavailability of poorly absorbed drugs (Mohammad et al., 2017; Dhumal et al., 2022). A self-assembly method of MF using phosphatidylcholine and chitosan has achieved a complete release of MF within 60 min for oral administration (Duyen and Duy, 2024). Self-assembled phospholipidic nanomicelles of MF, co-delivered with vitamin E, enhanced the cytotoxicity and cellular uptake on MCF-7 and MDA-MB-231 breast cancer cell lines, resulting in a higher and faster uptake by the cells (Khurana et al., 2018). Carbon dots (CDs) have been developed as organic carriers, but they also have photoluminescent and chemical structures that may be adapted for several pharmaceutical applications (Magdy et al., 2023). A water-soluble CD nanoemulsion containing MF (MF-CD) has demonstrated an increase in pharmacokinetic parameters, especially showing a 1.6-fold increase in the area under the curve for the maximum blood concentration in normal rats. This finding presented a novel approach for developing MF formulations with enhanced bioavailability (Kong et al., 2024).

A specific study investigated polyethylene glycol linked to carbon nanotubes (PEG-CNTs) as carriers for methotrexate (MT) in brain cancer treatment (Harsha et al., 2019). Cytotoxicity studies conducted on the U-87 brain cancer cell line showed a 1.28-fold decrease in the IC50 dose compared to MTX alone. This suggests that PEG-CNTs may be a promising approach to enhance the antitumor effects of MTX. Nanoparticles of MTX combined with hyaluronic acid (HA) have been synthesized using a self-assembly method to reduce the toxicity of MTX in cancer treatment (Wang et al., 2023). The MF-HA-MTX nanoparticles specifically inhibited the K7 cancer cell line while exhibiting lower toxicity compared to traditional MTX chemotherapy. A similar study by Meng et al. (2019) reported the assembly of an ionic peptide (RADA16-I) with MTX. The RADA16-I-MF-MTX nanoparticles demonstrated a significantly greater inhibition of colorectal adenocarcinoma cells (DLD-1) and colon cancer cells (KYSE 30) compared to MTX alone after 24, 48, and 72 h. MF has also been used as a carrier for delivering antitumor drugs (Zheng et al., 2025). The MF carrier consists of four components: i. a modulator for the tumor’s inflammatory environment, ii. an inducer of ferroptosis, iii. a tumor-penetrating agent, and iv. MF itself, which together form a self-assembled MF amphiphile that incorporates the antitumor drug (paclitaxel). The anticancer efficacy of the MF-carried formulation against paclitaxel-resistant breast tumors was confirmed in both *in vitro* and *in vivo* studies, demonstrating the effectiveness of this new cancer treatment approach.

In summary, organic carriers have been widely used to enhance the biological effects of MF. Among these, NLCs appear to be the most promising option for improving MF bioavailability when combined with specific tumor-targeting agents. Additionally, CyDs, CDs, and carbon nanotubes have been recently introduced as organic carriers for MF. These carriers have demonstrated favorable properties for cancer treatment due to their low toxicity in cancer cell lines.

### 5.2 Inorganic carriers

Gold nanoparticles (AuNPs) with anti-microbial, anti-viral, and anti-tumor properties have gained attention in prostate cancer, as summarized in a recent review by Mitri et al. (2023). In addition to their cytotoxic effects on cancer cell lines, particularly prostate cancer cells, AuNPs can effectively target tumor cells by delivering antibodies and ligands that specifically eliminate prostate tumors (Khoobchandani et al., 2021). The synthesis of MF-loaded gold nanoparticles (MF-AuNPs) and their effects on the MCF-10A breast cancer cell line have been reported by Patra et al. (2018). Their study noted the cleavage of the C-C bond of the pyranose moiety along with the oxidation of the phenolic hydroxyl groups (C1, C3, C6, and C7) during the formation of MF-AuNPs. Furthermore, Aboyewa et al. (2021) indicated that MF-AuNPs could be beneficial in the cotreatment of colorectal cancer when used alongside doxorubicin, showing effectiveness in the Caco 3 cancer cell line and HT-29 (colorectal adenocarcinoma), as well as in MDA-321 (breast cancer) cell lines (Aboyewa et al., 2022).

Radioactive gold (^198^Au) nanoparticles loaded with MF (MF-^198^AuNPs) have been shown to enhance radiotherapy for prostate cancer (Al-Yasiri et al., 2017). The intratumoral delivery of MF-^198^AuNPs demonstrated that over 80% of the injected dose remained in prostate tumors for up to 24 h. Additionally, there was a five-fold reduction in tumor volume after 3 weeks of treatment compared to the control group, which received a saline solution. Moreover, MF-^198^AuNPs have led to efficient endocytosis of prostate tumor cells, via the MF pyranose moiety, in SCID mice implanted with prostate tumor (PC-3) xenografts (Katti et al., 2017; Katti et al., 2018).

Zinc oxide (ZnO) has recently been utilized as an encapsulating agent for various antitumor drugs due to its cytotoxic effects associated with compounds produced during the synthesis of ZnO nanoparticles (Hamrayev et al., 2020; Razura-Carmona et al., 2022; 2023; Wang Y. et al., 2022; Missier et al., 2024; Shubha et al., 2024). Extracts from *Mangifera indica* (mango) containing MF have been encapsulated in ZnO nanoparticles and tested against the A549 lung cancer cell line (Rajeshkumar et al., 2018). The cytotoxic effect of the ZnO-microencapsulated mango extract increased with higher concentrations of the formulation and was comparable to that of the positive control, cyclophosphamide, at lower doses.

Mesoporous silica (Syloid^®^ XDP 3050) has been reported to form MF complexes following rotary mill mixing with MF in a 1:1 ratio (Baán et al., 2019), leading to enhanced water solubility; however, no biological data were provided. MF magnetic microspheres (MF-MG-MS) have been identified as potential carriers for cancer treatment (Xiao et al., 2021). The MF-MG-MS carrier was synthesized using iron acetylacetonate in phenyl ether, incorporating oleic acid and oleylamine. Additionally, copolymers (PCL-PEG-PCL) were created through the ring-opening of ε-caprolactone, and MF-MG-MS were produced by the solvent diffusion method.

### 5.3 Polymer-based carriers

Polymer-based carriers are micro- or nano-particle spherical matrices that deliver bioactive molecules. These carriers can be created using one or more types of polymers (Lu et al., 2021). They have been utilized in various applications, including bone and cartilage tissue engineering (Zhou et al., 2023), cancer treatment formulations (Yan et al., 2018), ocular drug delivery (Naik et al., 2020), insulin delivery (Mansoor et al., 2019), and vaccine delivery systems (Lambricht et al., 2017), among others. Various polymer-based carriers have been employed to enhance the bioavailability of MF, as discussed in a review by Morozkina et al. (2021).

Chitosan, a polysaccharide derived from acid hydrolysis of chitin, and chitosan-modified polymers have attracted the scientific community as drug carriers for several applications (Ghaz-Jahanian et al., 2015; Demeyer et al., 2021; Saeedi et al., 2022; Goyal et al., 2024; Athipornchai et al., 2024; Lv et al., 2024). Samadarsi and Dutta (2020) utilized MF-chitosan nanoparticles (MF-Chi-NPs) with tripolyphosphate as a crosslinker to enhance the antioxidant effects of MF. In addition to exhibiting a greater free radical scavenging effect, as compared to free MF, the MF-Chi-NPs demonstrated a synergistic effect on the antioxidant enzymes catalase and peroxidase. This led to improved protection against protein oxidation and enhanced inhibition of lipid peroxidation. The effectiveness of MF carboxymethyl chitosan on the MG63 osteosarcoma cell line has been shown to inhibit cell growth, with IC50 values ranging from 7.8 to 15.6 μg/mL. In comparison, MF required significantly higher concentrations (Yusri et al., 2020). Additionally, MF has been incorporated into alginate-grafted N-succinyl chitosan (MF-Chi-NSC) to lower glucose, cholesterol, and triglyceride levels (Wang Y. et al., 2022). *In vivo* experiments demonstrated a reduction in glucose levels from 300 to 90 mg/mL with MF-Chi-NSC (300–180 mg/mL with MF); cholesterol levels decreased by approximately 37% (compared to 1%–36% with MF), and triglyceride levels dropped by around 60% (10%–40% with MF).

The research conducted by Pipattanawarothai et al. (2019) investigated the loading of MF into various blending systems, including binary systems composed of polyvinyl alcohol (PVA) and chitosan (CHI), as well as ternary systems that combine PVA, CHI, and gelatin. The study revealed that MF can form hydrogen bonds with the amide groups of chitosan and the hydroxyl groups of polyvinyl alcohol in homopolymer matrices. Notably, MF exhibited a stronger tendency to form intermolecular hydrogen bonds with the hydroxyl groups of chitosan compared to those of polyvinyl alcohol. Consequently, as the content of chitosan in the polymer-based carrier increases, the release of MF decreases.

The enhancement of brain bioavailability for MF has been achieved using polylactic-glycolic acid (PLGA) nanoparticles coated with polysorbate 80, administered via intranasal delivery in rats through an ischemia-induced model (Ahmad et al., 2024). The absorption of MF was higher than 80%, with a controlled release lasting 8 h. Additionally, MF-β-LG nanoparticles were formulated with β-lactoglobulin using tripolyphosphate as a cross-linker, demonstrating an 80% release in simulated colonic fluid within 8 h and only 9% release in simulated gastric fluid. This suggested that these nanoparticles could be a promising system for targeted MF delivery in oral formulations (Samadarsi and Dutta, 2019). However, there is a risk that the nanoencapsulation with β-lactoglobulin could reduce the biological properties of MF, which may affect its oral bioavailability.

Targeted polymeric nanoparticles have been developed to deliver MF with a high loading content of 6.86% ± 0.60%. These nanoparticles demonstrate excellent blood circulation and exhibit missile-like delivery to the pancreas (Wang M. et al., 2022). A pancreas-targeting agent, GLP-1, was immobilized on the copolymer polyethylene glycol-polycaprolactone (PEG-PCL) to create GLP-1-PEG-PCL (GLPP). These nanoparticles were self-assembled with MF, resulting in MF-GLPP nanoparticles that exhibited a higher concentration in the pancreas compared to free MF formulations *in vivo*. MF particles loaded with PGE have been evaluated concerning the inhibition of the enzyme α-glucosidase, which showed a more effective inhibition, around 95.42%, when compared to the free-form MF (90.42%) (Bezerra et al., 2019).

Recently, there has been a review of alternatives to forming macromolecular organic carriers with an inhibitor to enhance cell permeability (Skwarecki et al., 2020). Once these conjugates are internalized into the cell, either through direct translocation or endocytosis, they can release the active compound and target intracellular sites. Polymer-based carriers for the bioactive compound have demonstrated their high structural versatility, making them an appealing option for delivery. Modifying these carriers using homopolymers, copolymers, peptides, or proteins can facilitate specific targeting of therapeutic or diagnostic active sites. This targeting potentially increases the efficacy and sensitivity of the treatment. Table 2 summarizes the MF-drug carriers that enhance MF’s water solubility and permeability.

**TABLE 2:** Several types of mangiferin (MF) carriers to enhance its water solubility and cell permeability.

Type	Sub-type	Carrier	Dose	Enhanced effect	References
Organic Carriers	Lipidic	888ATO + miglyol	2 μM	↑Antioxidant effect in ocular delivery	Santonocito et al. (2022)
		Phospholipon 90H	0.1 mM	↑Antioxidant effect	Telange et al. (2021)
		Phospholipon90G + compritol + Labrafil M2125	100 μM	↑Cytotoxicity on CaCo 3 cells	Khurana et al. (2017a)
		Labrafil M 2130CS + Tween 80	0.2 μM	↑Antidiabetic	Foudah et al. (2024)
		DSPE + Transferrin	7.5 μM	↑Cytotoxicity on A549 cells	Zhou et al. (2023)
	Nanoemulsion	Phosphatidylcholine + chitosan	NA	↑MF release (100%)	Duyen and Duy (2024)
		Phospatidylcholine + sodium glycolate	NA	↑Cytotoxicity on CaCo 2 cells	Thiengkaew et al. (2021)
		Phospholipon90G + vitamin E-TPGS	4.4 nM	↑Cytotoxicity on MCF-7 cells	Khurana et al. (2018)
		Carbon dots	>1.5 μM	↓Toxicity in human normal cells (H7-22)	Kong et al. (2024)
	Amphiphilic carrier	Mangiferin	NA	↓Drug chemoresistance in cancer treatment	Zheng et al. (2025)
	Carbon nanotubes	Polyethylene glycol	8–26 μM	↑Cytotoxicity on U-87 cells	Harsha et al. (2019)
Inorganic carriers	Nanoparticles	Gold	NA	↑Targeted delivery for prostate cancer	Mitri et al. (2023)
			0.25 mM	↓Toxicity in human normal cells (MCF-10A)	Patra et al. (2018)
			2.4 mM	↑Cytotoxicity of DOX on U87 cells	Aboyewa et al. (2021)
			2.4 mM	↑Cytotoxicity in breast cancer	Aboyewa et al. (2022)
Inorganic carriers	Nanoparticles	Radioactive gold	5–15 μM	↑Cytotoxicity on PC-3 cells	Al-Yasiri et al. (2017)
	Metal oxides	Zinc oxide (ZnO)	NA	↑Cytotoxicity on A549 cells	Rajeshkumar et al. (2018)
	Mesoporous silica	Syloid^®^XDP3050	NA	↑Water solubility	Baán et al. (2019)
	Mesoporous silica	SBA15	NA	↑Water solubility	Pontes-Silva et al. (2017)
	Microspheres	Magnetic iron III	NA	↑Cytotoxicity on cancer cell lines	Xiao et al. (2021)
Polymer-based carriers	Polysaccharide	Chitosan	NA	Several biological effects	Athipornchai et al. (2024) Demeyer et al. (2021) Samadarsi and Dutta (2020)
		Chitosan-PVA	NA	↑Water solubility	Pipattanawarothai et al. (2019)
		Chitosan-PVA-gelatin	NA	↑Water solubility	Pipattanawarothai et al. (2019)
		N-succinyl-alginate-chitosan	23.6 μM	↑Hypoglycemic activity	Wang et al. (2022b)
		Carboxymethyl-chitosan	18–36 μM	↑Cytotoxicity on MG63 cells	Yusri et al. (2020)
	Copolymers	PLGA-Polysorbate 80	10–20 μM	↑MF brain bioavailability	Ahmad et al. (2024)
		PLGA-ZnO		↑Cytotoxicity on HepG2 cells	Fabián et al. (2023)
		GLP-1-PEG-PCL	2 μM	↑Hypoglycemic activity\	Wang et al. (2022b)
	Protein	β-lactoglobulin	900 μM	Colonic control release	Samadarsi and Dutta (2019)
		Ovoalbumin	NA	↑Anti-diabetic effect	Chen and Zhen (2022)

Legend: PVA: polyvinyl alcohol; PLGA: polylactic globulinic acid; PEG: polyethylenglicol; PCL: polycaprolactone; GLP-1: Pancreas-targeting agent; DSPE: 1,2-diestearoyl-sn-glycero-3-phosphoethanolamine; TPGS: α-Tocopheryl polyethylene glycol succinate; MF: mangiferin; DOX: doxorubicin; NA: not applicable.

## 6 Future perspectives

**FIGURE 10:**
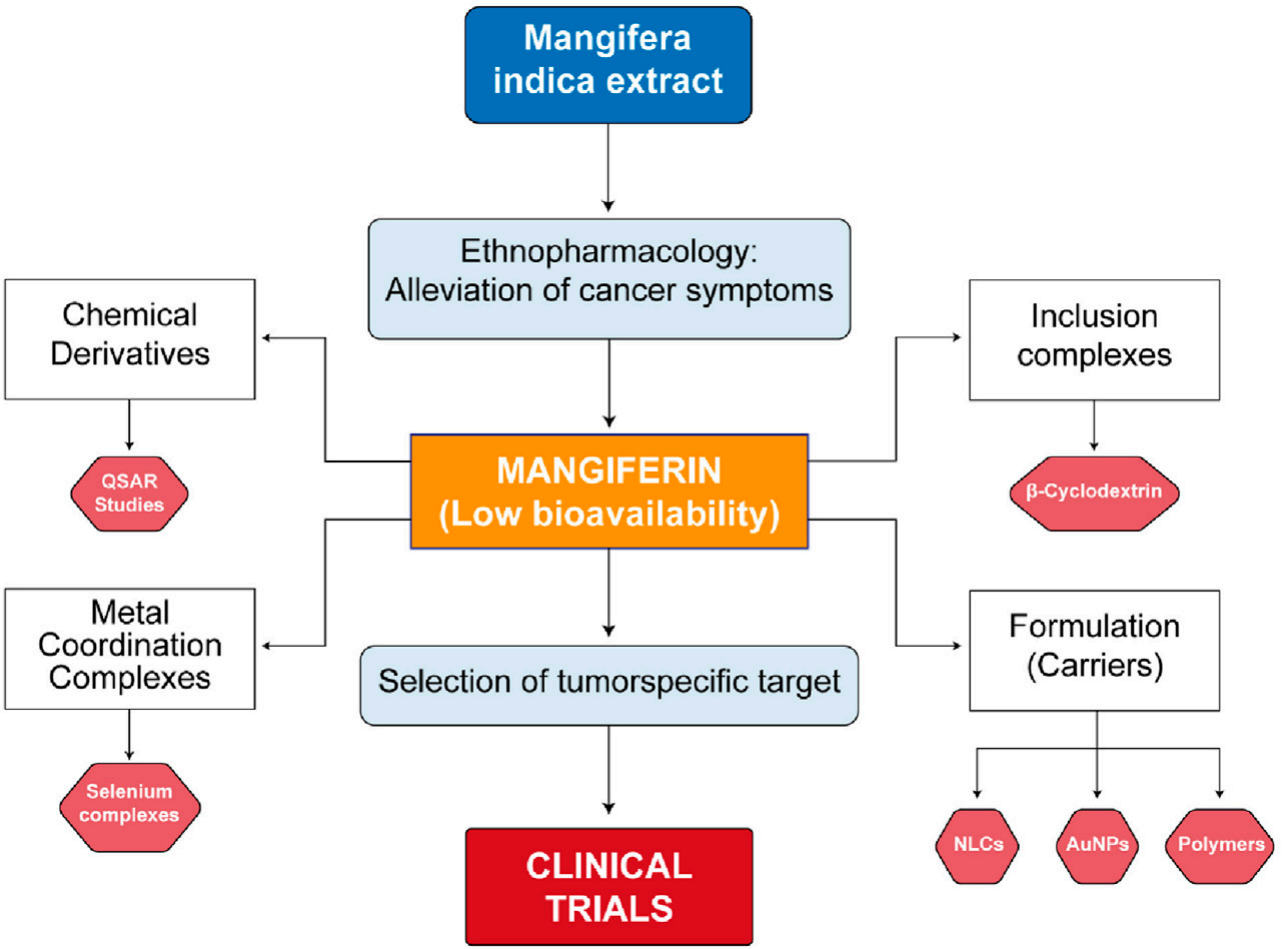
Diagram of main challenges for improving mangiferin solubility and bioavailability for cancer treatment. (QSAR: Quantitative Structure-Activity Relationships, NLCs: Nanolipid Carriers, AuNPs: Gold Nanoparticles).

Future research on the synthesis of chemical derivatives, coordination metal complexes, and carriers for the formulation of MF in cancer treatment should focus on several key directions to enhance its therapeutic efficacy and bioavailability (Baghel et al., 2024). Firstly, the development of novel synthetic methods to create MF derivatives with improved physicochemical properties could yield compounds with higher potency against various cancer types. Derivatives optimization for cancer treatment should explore QSAR modelling for specific cancer targets using adequate structure descriptors. Within the metal coordination complexes with MF as ligand, the use of selenium as the central nucleus has shown better results as compared to other metals. The challenge is to conduct clinical trials to prove the initial results in clinical practice. Evaluating the synergistic effects of MF when combined with existing chemotherapy agents may also provide insights into multi-modal treatment strategies that can overcome resistance mechanisms. The exploration of advanced nanocarrier systems—such as liposomes, polymeric nanoparticles, and dendrimers—could facilitate targeted delivery of MF to tumor-specific sites, minimizing systemic toxicity and maximizing therapeutic effects. Implementing strategies to enhance the solubility and stability of MF is crucial to ensure adequate bioavailability. Furthermore, *in vivo* studies and clinical trials should be prioritized to assess the safety and effectiveness of these formulations in cancer patients. Overall, a multi-faceted approach incorporating synthetic chemistry, material science, and pharmacology will be essential to fully realize the potential of MF as an effective anti-cancer agent. A summary of future directions in research on MF’s challenges for cancer treatment is shown in Figure 10.

## 7 Conclusion

The most significant advantage of MF derivatives, complexes, and carriers for cancer treatment is their improved bioavailability, leading to higher cell penetrability and thus higher cytotoxicity on cancer cells. Glycoside derivatives and metal coordination complexes have shown great promise among the reviewed MF derivatives for cancer treatment. Further research is needed to explore the advantages of MF-Se (IV) complexes, which potentially enhance both bioavailability and bioactivity, particularly considering the biological importance of selenium as a cofactor for endogenous antioxidant enzymes. Among various matrices, β-Cyclodextrin has emerged as the most effective for delivering MF due to its toroidal structure and biocompatibility. However, research on the antitumor effects of MF derivatives, complexes, and carriers for cancer treatment remains limited, both *in vivo* and in clinical settings. MF-based Nanostructured Lipid Carriers (NLCs), Gold Nanoparticles (AuNPs), and Polymer-based Nanoparticles (PNPs) have been more extensively investigated and have proven to be viable technological alternatives to address the water solubility challenges associated with MF. Comparative studies efficacy of all these carriers for delivering MF in cancer treatment are needed. All of these strategies to enhance MF’s bioavailability and cell penetration lay the groundwork for future drug development in cancer research.
